# Evaluating the effectiveness of localized control strategies to curtail chikungunya

**DOI:** 10.1038/srep23997

**Published:** 2016-04-05

**Authors:** Martial L. Ndeffo-Mbah, David P. Durham, Laura A. Skrip, Elaine O. Nsoesie, John S. Brownstein, Durland Fish, Alison P. Galvani

**Affiliations:** 1 Department of Epidemiology of Microbial Disease, Yale School of Public Health, New Haven, CT, USA; 2 Children’s Hospital Informatics Program, Boston Children’s Hospital, Boston, Massachusetts, USA; 3 Department of Ecology & Evolutionary Biology, Yale University, New Haven, CT, USA

## Abstract

Chikungunya, a re-emerging arbovirus transmitted to humans by *Aedes aegypti* and *Ae. albopictus* mosquitoes, causes debilitating disease characterized by an acute febrile phase and chronic joint pain. Chikungunya has recently spread to the island of St. Martin and subsequently throughout the Americas. The disease is now affecting 42 countries and territories throughout the Americas. While chikungunya is mainly a tropical disease, the recent introduction and subsequent spread of *Ae. albopictus* into temperate regions has increased the threat of chikungunya outbreaks beyond the tropics. Given that there are currently no vaccines or treatments for chikungunya, vector control remains the primary measure to curtail transmission. To investigate the effectiveness of a containment strategy that combines disease surveillance, localized vector control and transmission reduction measures, we developed a model of chikungunya transmission dynamics within a large residential neighborhood, explicitly accounting for human and mosquito movement. Our findings indicate that prompt targeted vector control efforts combined with measures to reduce transmission from symptomatic cases to mosquitoes may be highly effective approaches for controlling outbreaks of chikungunya, provided that sufficient detection of chikungunya cases can be achieved.

Since the 1953 identification of chikungunya in Tanzania^1^, numerous epidemics have been reported in Africa^2^,^3^, Asia^3^,^4^,^5^, Europe^6^,^7^ and the Americas^8^, ranging in magnitude from a few hundred to over a million cases. Until recently, chikungunya was regarded as an exclusively tropical disease transmitted principally by *Ae. aegypti*^9^. However, the introduction and subsequent spread of *Ae. albopictus* into temperate regions, combined with the recent evolution of chikungunya towards elevated transmissibility in *Ae. albopictus*, has exacerbated the risk of temperate chikungunya outbreaks^8^,^10^. In December 2013, *Ae. aegypti* - transmitted chikungunya was initially introduced on the island of St. Martin from which it disseminated throughout the Caribbean, threatening both mainland Latin America, where *Ae. aegypti* mosquito is widespread, and the southern United States, where both *Ae. aegypti* and *Ae. albopictus* are prevalent^11^,^12^,^13^,^14^. As of February 2016, chikungunya has been reported in at least 42 countries across the American continent, including the United States and its territories (i.e. Florida, Puerto Rico, and the U.S. Virgin Islands)^15^.

Chikungunya is characterized by the sudden onset of fever and joint pain, which are often incapacitating^16^. The acute febrile phase can become chronic, with debilitating joint pains that can persist for several weeks or even months^3^. Although the disease is fatal in fewer than one in 10,000 cases, chikungunya can nonetheless impose a high health burden and societal cost^17^,^18^,^19^,^20^. The recent introduction of chikungunya in the Western Hemisphere poses a serious threat to public health, particularly in countries where both *Ae. aegypti* and *Ae. albopictus* are endemic^8^,^11^,^21^. In the absence of a vaccine, containing chikungunya outbreaks is challenging and relies on promptly interrupting transmission^22^. Most cases of chikungunya are symptomatic and can be accurately diagnosed^23^,^24^. Reverse transcriptase-polymerase chain reaction or serology has been used to confirm introductory cases in new areas, but are generally regarded as too expensive to be routinely employed in ongoing surveillance^3^,^25^. Here we evaluate the effectiveness of perifocal mosquito control around houses with detected cases as well as transmission reduction measures that decrease contact between infected humans and mosquitoes, including bed nets and mosquito repellents to reduce contact between symptomatic individuals and mosquitoes.

To assess the effectiveness of interventions against chikungunya, we developed a stochastic vector-borne transmission model for the spread of chikungunya within a residential neighborhood. Our model takes into account the impact of household transmission of chikungunya as well as human and mosquito movement between houses. We parameterized the model using epidemiological, entomological, clinical, and human movement data. For both *Ae. aegypti* and *Ae. albopictus*, we evaluated the effectiveness of intervention strategies for mitigating the spread of a chikungunya outbreak. In contrast to the contention that little can be done to contain chikungunya^11^, we found that vector control and reducing contact between symptomatic humans and mosquitoes can interact synergistically to effectively contain chikungunya. Specifically, we found perifocal mosquito control targeting residences of infected individuals to be essential in reducing mosquito infection, while effective transmission reduction measures of patients after diagnosis should prevent further infection. Implemented together, these interventions can substantially reduce the attack rate of chikungunya and contain outbreaks.

## Methods

To simulate viral transmission in a residential neighborhood, we developed a household-level spatial stochastic model of chikungunya transmission, explicitly modeling individual human and mosquito movement and exposure. We ran our continuous-time model for one year using the Gibson-Bruck adaptation of the Gillespie algorithm^26^. Using this algorithm, we iteratively sampled and executed randomly chosen events, including entomological events, such as the movement of a mosquito to an adjacent house, or epidemiological events, such as the acquisition or clearance of chikungunya infection by a human. After each event was executed in the simulation, the transition probabilities for all events were updated to reflect the new state of the system.

We ran our model on a two-dimensional square lattice with 20 × 20 sites, representing a neighborhood of 400 houses populated with four residents per house^27^,^28^. As our time scale of interest was a one-year period, we assumed the human population to be constant with no human mortality, and the mosquito population dynamics to be governed by a household-level carrying capacity.

We assumed that humans divide their time between their homes and other houses, with the likelihood of the latter depending on the distance of other residences away from their homes, as informed by an empirical study^29^. Each house *i* was assigned a number of contact houses *n*_*i*_ sampled from an exponential distribution with mean of 6.26 29. We assumed that, on average, an individual in house *i* spends a fraction 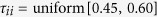
30 of time at home. The remaining time was spent in one of the *n*_*i*_ contact houses, chosen randomly and each assigned an exponentially distributed weight *τ*_ij_ and normalized such that 
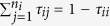
.

Individuals were designated as susceptible, exposed, infected (pre-symptomatic), infected (70–90% of whom were asymptomatic), or recovered from chikungunya, at rates parameterized from epidemiological, clinical and entomological studies (Table 1). The transmission of chikungunya from infectious mosquitoes in house *j* to humans from house *i* was modeled as 
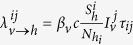
, where 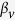 is the transmission rate from mosquito to human, *c* is the biting rate, 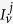 is the number of infectious mosquitoes in house *j*, 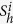 is the number of susceptible humans in house *i*, *N*_*hi*_ is the number of humans in house *i*, and *τ*_*ij*_ is the proportion of time spent in house *j* by the individual from house *i*. After an incubation period ranging from two to six days, exposed humans become pre-symptomatically infectious for two days^3^,^24^,^31^,^32^. Following this pre-symptomatic period, a proportion *κ* of infectious humans become symptomatic while the rest remain asymptomatic.

### Mosquito dynamics and epidemiology

We parameterized the daily rate of mosquito movement between houses from a field study (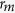 = 0.044 per day)^33^. We specified a household-level carrying capacity for each house, and sampled the carrying capacity independently for each house from an exponential distribution of mean 7.8 mosquitoes^34^. For simplicity, we assumed periodic boundary conditions for mosquito movement. In the absence of interventions, humans were bitten by mosquitoes at a fixed daily rate within every home^35^,^36^,^37^,^38^. As described below, we modeled transmission-reduction measures, such as bed nets, in terms of a reduced biting rate. We accounted both for the bites on infectious human hosts that could result in infection of a susceptible mosquito and, conversely, for the bites from infectious mosquitoes that could infect susceptible humans.

**Figure 1:**
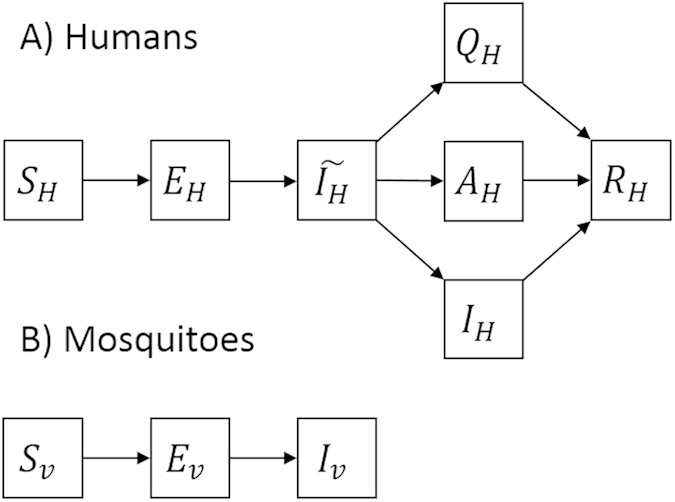
Model diagram. The model consisted of humans (H) and mosquitoes (V). Humans could be susceptible (*S*_*H*_), exposed (*E*_*H*_), pre-symptomatically infected (*Î*_*H*_), asymptomatically infected (*A*_*H*_), symptomatically infected (*I*_*H*_), quarantined (*Q*_*H*_), or recovered (*R*_*H*_). Mosquitoes could be susceptible (*S*_*V*_), exposed (*E*_*V*_), or infected (*I*_*V*_).

Each mosquito was designated as susceptible, exposed, or infected with chikungunya. The transmission of chikungunya from asymptomatic infectious humans residing in house *i* to mosquitoes in house *j* was given as 
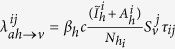
, where *β*_*h*_ is the transmission rate from human to mosquitoes, 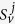 is the number of susceptible mosquitoes in house *j*, 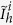 is the number of pre-symptomatic infectious humans from house *i*, and 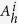 is the number of asymptomatic infectious humans from house *i*. Based on empirical data of humans symptomatic with chikungunya, we assumed a 50–80% reduction in mobility of symptomatic infected humans^23^,^39^,^40^. The corresponding transmission from symptomatic humans in house *i* to mosquitoes in house *j* was given as 
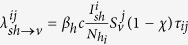
 for *i* ≠ *j* and 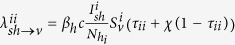, where *χ* is the reduction in mobility of symptomatic individuals and 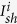 is the number of symptomatic infectious humans from house *i*. Exposed mosquitoes become infectious after an extrinsic incubation period and remain infectious until they die. We separately considered *Ae. aegypti* and *Ae. albopictus* mosquito populations, parameterized with respective epidemiological and entomological data (Table 1). We provided a model diagram for illustration (Fig. 1).

### Calibration

We evaluated the effectiveness of containment intervention strategies for mitigating the spread of an emerging chikungunya outbreak. We used the basic reproduction number (*R*_0_) as a measure of the likelihood of disease outbreak and intensity of spread through the population. To calculate *R*_0_, we simulated the number of secondary cases of human infections that resulted from mosquitoes that were directly infected by an initial human case in a population of fully susceptible humans and mosquitoes. By simulating 10,000 iterations of our stochastic model, we generated a probabilistic distribution of secondary cases likely to arise from a single infection, with mean *R*_0_ 41,42. The transmission parameter 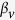 was separately calibrated for both *Ae. aegypti* and *Ae. albopictus* by fitting the simulated distribution of *R*_0_ against empirical estimates from previous and current chikungunya outbreaks^31^,^43^. These chikungunya *R*_0_ values were estimated for the strain of chikungunya transmitted by *Ae. albopictus* during the 2004–2005 epidemic in Réunion Island^31^ and the 2007 epidemic in Italy^6^, and for the strain of chikungunya transmitted by *Ae. aegypti* for the 2005–2006 epidemic in India^44^ and ongoing outbreaks in the Caribbean^43^. With the exception of 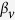, which we calibrated, the model parameters were obtained from literature (Table 1). We illustrate this method of calculation in Supplementary Material (Fig. S1).

Containment measures for chikungunya include perifocal space spraying (fogging) of insecticides in perimeter radius around houses with detected cases, indoor residual spraying^22^,^45^,^46^, the application of mosquito repellents on symptomatic individuals and the use of bed nets for patients in their homes^3^,^45^,^46^. We considered intervention strategies combining different levels of: 1) perifocal and indoor residual spraying in houses with reported cases and directly neighboring houses and 2) transmission reduction measures, specifically bed nets and/or mosquito repellents, for diagnosed individuals to reduce subsequent mosquito bites, both to household members and neighbors. We assumed that vector control only affected mosquitoes that were present in and around treated houses, with the efficacy of vector control incorporated into the model as the probability of mosquito mortality from perifocal or indoor residual spraying. The efficacy of transmission reduction measures for symptomatic individuals was incorporated by reducing the contact rate between humans and mosquitoes which results in a reduction of transmission rates of human-to-mosquito and mosquito-to-human. With the implementation of these intervention measures, the transmission from symptomatic humans to mosquitoes was given as 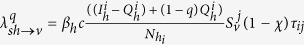, where 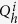 is the number of symptomatic humans using transmission reduction measures, with 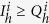, and *q* is their efficacy for reducing contact with mosquitoes. We evaluated the effectiveness of intervention strategies for mitigating a chikungunya outbreak over varying levels of disease surveillance, defined as the probability of case detection. Effectiveness of interventions was measured in terms of the attack ratio, defined as the proportion of individuals who are infected over the course of the epidemic. The attack ratio was computed by averaging the proportion of individuals infected during the simulated one year epidemic period over the 1000 iterations of our model. To account for differing efficacies of and compliance to the control measures, we varied the efficacy of the intervention strategy for detecting new cases of chikungunya, removing adult mosquitoes through vector control, and reducing biting rate on symptomatic individuals to whom transmission reduction measures are targeted. We considered different levels of chikungunya transmissibility (R_0_ = 2, 4, 6), efficacies of vector control for killing adults mosquitoes (ranging from 10 to 90%), and sensitivities of surveillance measures for detecting new cases of symptomatic human infection (low (30% efficacy), intermediate (50% efficacy), high (80% efficacy)). A flowchart outlines the steps of our analysis (Fig. S2).

## Results

**Figure 2:**
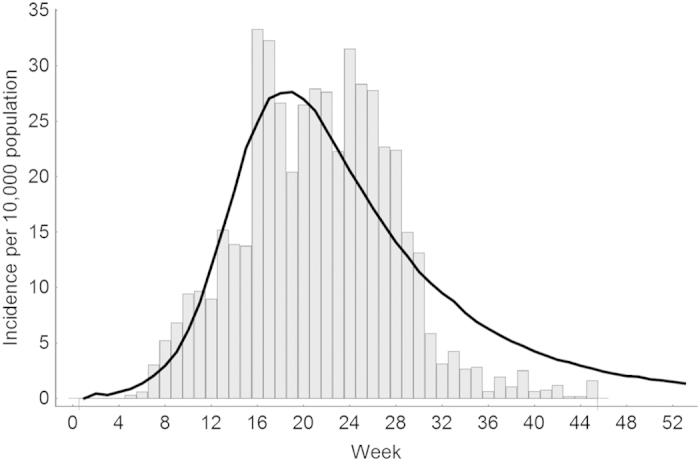
Model validation. We compare the time series of chikungunya cases generated by our model for *R*_0_ =4 against empirical data of chikungunya cases reported in Dominica during the 2013–2014 outbreak^58^. The solid line represents the average realization of our model while the histogram bars represent the empirical data from Dominica.

**Figure 3:**
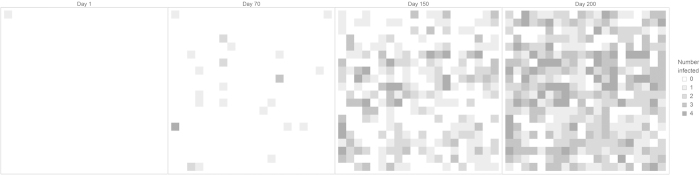
Simulated spatiotemporal dynamics of chikungunya cases. Four snapshots of spatial distribution of cases are shown at time t = 0, 70, 150, 200 days following the index case.

For *Ae. aegypti*, the transmission parameter 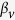 was estimated to be 0.06 when *R*_0_ equals 2, 0.14 when *R*_0_ equals 4 and 0.24 when *R*_0_ equals 6. For *Ae. albopictus*, the transmission parameter 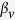 was estimated to be 0.1 for *R*_0_ equals 2, 0.22 for *R*_0_ equals 4 and 0.36 for *R*_0_ equals 6. We validated the time series dynamics of our model against empirical data from the 2013–2014 chikungunya outbreak in Dominica (Fig. 2), and illustrated the spatiotemporal dynamics with spatial snapshots of cases throughout the modelled residential neighborhood (Fig. 3).

**Figure 4:**
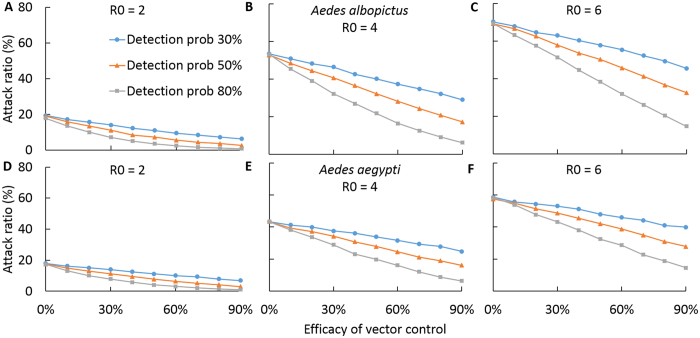
Effect of vector control for reducing the attack ratio of chikungunya. Average attack ratio of chikungunya for ranges of vector control efficacies and disease surveillance sensitivities for different *R*_0_ values (**A**,**D**) *R*_0_ = 2, (**B**,**E**) *R*_0_ = 4, (**D**,**F**) *R*_0_ = 6. We compared (**A**–**D**) *Aedes albopictus* and (**D**–**F**) *Aedes aegypti* as disease transmission vectors.

We evaluated the effectiveness of a reactive perifocal vector control strategy for curtailing chikungunya, which consists of insecticide spraying in perimeter radius around houses with infected individuals, as well as indoor residual spraying. We found that perifocal vector control has the potential to be effective for curtailing the spread of a chikungunya outbreak. However, its effectiveness is sensitive to the efficacy of surveillance measures for identifying new cases (Fig. 4). We showed that for a low 30% probability of detecting new symptomatic cases, even a highly efficacious vector control measure with 90% efficacy would have only a marginal reduction of the epidemic attack ratio (Fig. 4). However, for a detection efficacy of 80%, vector control has the potential to substantially reduce the epidemic attack ratio even when the vector control has an intermediate efficacy of 60% (Fig. 4).

**Figure 5:**
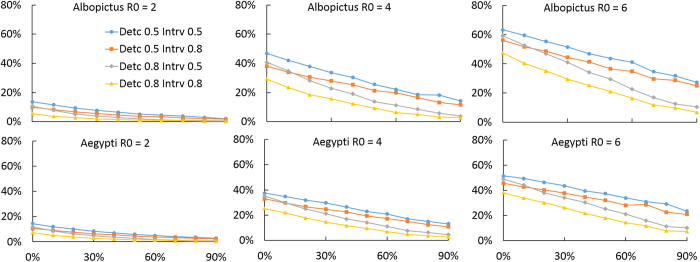
Effect of combined vector control and transmission reduction measures for reducing attack ratio of chikungunya. Average attack ratio of chikungunya for ranges of vector control efficacies, disease surveillance sensitivities, and efficacies of transmission reduction measures for symptomatic individuals for different *R*_0_ values (**A**,**D**) *R*_0_ = 2, (**B**,**E**) *R*_0_ = 4, (**D**,**F**) *R*_0_ = 6. We compared (**A**–**D**) *Aedes albopictus* and (**D**–**F**) *Aedes aegypti* as disease transmission vectors.

We evaluated the effectiveness in terms of reducing the attack ratio of chikungunya of an intervention strategy combining perifocal vector control with measures that decrease mosquito biting of infectious individuals. We found that such transmission reduction measures interact synergistically with vector control to greatly improve the effectiveness of transmission mitigation, especially when vector control has low efficacy (Figs 4 and 5). In low transmission intensity settings (e.g. *R*_0_ = 2), the combination of disease surveillance measures with at least 80% probability of detecting new cases, efficacious transmission reduction measures for symptomatic individuals with at least 80% reduction of contact between infected individuals and mosquitoes, and vector control measures with at least 50% efficacy in killing adult mosquitoes was found to reduce the epidemic attack ratio from 20%, without control, to less than 1% (Figs 4 and 5). In high transmission intensity settings (e.g. *R*_0_ = 6), the combined intervention was found to reduce the epidemic attack ratio from 60% down below 20% (Figs 4 and 5).

**Figure 6:**
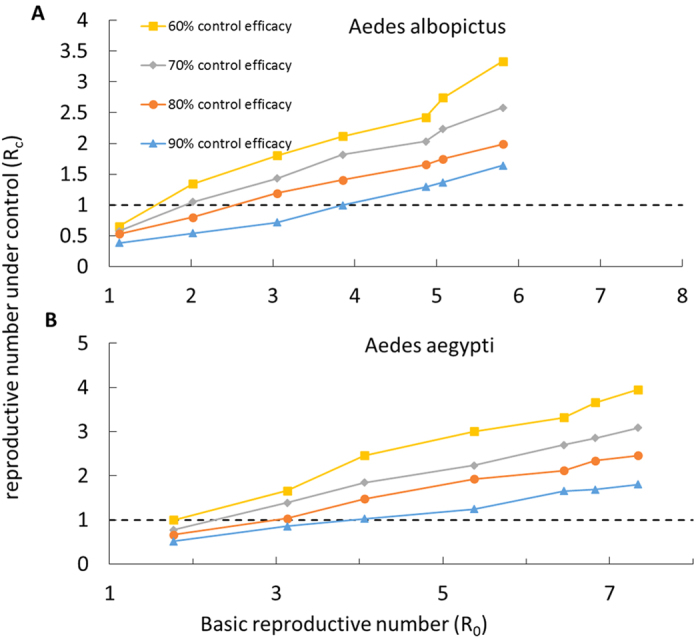
Relationship between Basic reproductive number (*R*_0_) and the Reproductive number under control (*R*_*c*_) for different efficacies of control implementation. Here, we assumed equal efficacies for cases detection, perifocal vector control, and disease reduction measures for symptomatic individuals. We compared (**A**) *Aedes albopictus* and (**B**) *Aedes aegypti* as vector species.

We also evaluated the impact of the combined intervention strategies on reducing the average number of secondary cases of human infections resulting from mosquitoes that were directly infected by the initial human case. We found that control measures combining surveillance, transmission reduction for symptomatic individuals, and perifocal vector control at an efficacy of at least 80% reduced initial *R*_0_ values of 3 − 6 to below 2, and initial *R*_0_ values of 3 and lower to below 1 (Fig. 6).

**Figure 7:**
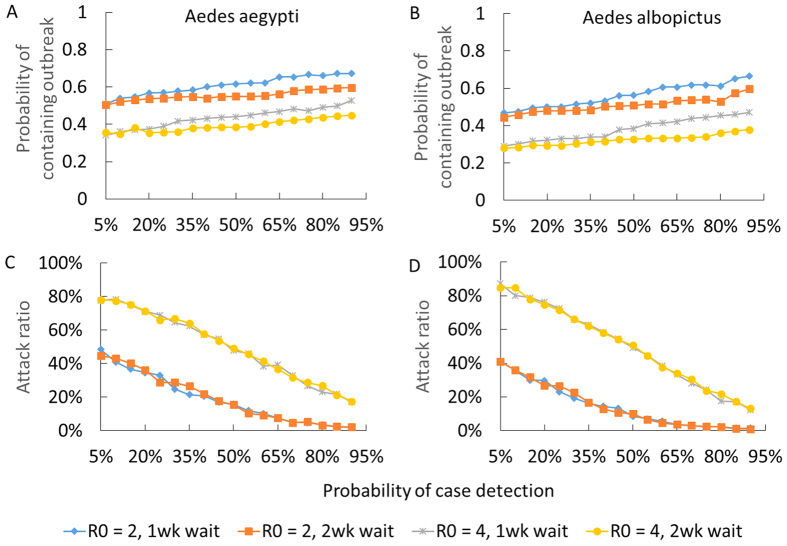
Impact of the efficacy of case detection on the chikungunya attack ratio when combined with efficacious vector control and transmission reduction around symptomatic individuals. The efficacies of vector control and transmission reduction measures were varied from 70 to 90%. We defined the effectiveness of controlling the outbreak as the probability that an index case will produce fewer than two secondary cases. The attack ratio was computed when an index case produced at least two secondary cases. We compared timing of intervention initiation with a one-week versus two-week delay from detection of the index case.

In our uncertainty analysis, we considered the impact of containment measures and of delays in implementing interventions upon chikungunya transmission. We evaluated the probability of preventing an outbreak, defined as the probability that an index case would not lead to less than one secondary case, when vector control and transmission reduction measures are implemented (Fig. 7A,C). We found that, even with a two week delay in initiating intervention, integrated efficacious vector control and transmission reduction for symptomatic individuals may be able to prevent a chikungunya outbreak (Fig. 7A,C). For example, control measures that combine surveillance with at least 70% case detection efficacy, transmission reduction measures and perifocal vector control, at an efficacy of at least 70% for each intervention, have at least a 60% probability of preventing a chikungunya outbreak when *R*_0_ is below 4 (Fig. 7A,C). However, if *R*_0_ is above 4, control measures fail to prevent an outbreak (Fig. 7A,C), although the magnitude of the resulting epidemic would vary substantially with the efficacy of disease surveillance measures (Fig. 7B,D). The probability of containing an outbreak decreases as both *R*_0_ and delay in initiating interventions increases (Fig. 7).

## Discussion

With nearly the entire Western Hemisphere presently at risk for chikungunya invasion, evaluating the effectiveness of measures for containment has become a public health priority^14^. Given that there are currently no vaccines nor specific treatments effective in reducing chikungunya transmission, it has been argued that little can be done to control chikungunya^11^. Using a spatial stochastic model for chikungunya transmission, we showed that, contrary to this assessment, perifocal vector control is capable of limiting the spread of chikungunya. We found that when vector control is integrated with transmission reduction around symptomatic individuals, the combined intervention strategy has the potential to interrupt disease transmission even after accounting for asymptomatic infection. We further found that timely implementation of combined interventions early in the outbreak is paramount. The strategies that we evaluated focus on targeted containment rather than widespread vector control, as has been successfully applied to contain the invasion and spread of dengue viruses in non-endemic areas^47^. Although the impact of perifocal vector control depends on the sensitivity of disease surveillance for detecting new cases of chikungunya, a high degree of accurate and swift clinical diagnosis has been achieved for cases in past epidemics^23^,^24^.

Previous chikungunya models have investigated the effectiveness of removing mosquito breeding sites^6^,^48^ and chemical control tools, such as adulticide and larvicide^48^,^49^, for controlling ongoing epidemics. However, these studies assumed homogeneous mixing of human and mosquito populations, and so could not accurately evaluate the effectiveness of reactive control strategies such as perifocal space spraying (fogging) of insecticides and indoor residual spraying^22^,^45^, which are spatially targeted strategies. Moreover, some of these studies have investigated the impact of control strategies through sensitivity analysis of model input parameters, rather than explicitly quantifying the intensity and speed of control efforts needed for curtailing disease spread^49^,^50^. Our study extends previous analyses by accounting for human and mosquito movements, focal application and impact of insecticide spray, as well as the integration of early vector control with transmission reduction measures for mitigating the spread of chikungunya. In so doing, our study evaluates optimal speed and efficacy of perifocal vector control and transmission reduction measures needed to curtail a chikungunya outbreak in a large residential neighborhood. Adherence to the use of transmission reduction measures such as bed nets, mosquito repellents, and long-sleeved garments may be promoted through health education that emphasizes the importance of these measures for reducing risk of infection for other household members^51^. Our model is applicable to tropical regions in which the continual abundance of mosquito populations gives chikungunya the potential to circulate throughout the year^52^,^53^. Climatic variables and other environmental factors may affect mosquito population dynamics and the risk of chikungunya outbreaks, especially in temperate regions^52^,^53^, where chikungunya transmission may vary seasonally^6^,^53^. Future study may take into account climatic factors, while the population social structure could be extended to account for age and gender heterogeneity, which may result in differential contributions to chikungunya transmission^54^. Previous studies have suggested that the effectiveness of spatially targeted insecticide spraying strategies for dengue control may be strongly affected by socially structured human movement^30^. Differences in the social structure of human movement, such as between rural versus urban settings, may impact the effectiveness of chikungunya perifocal vector control strategies. For example in this study, on the basis of empirical data from the city of Iquitos, Peru, we assumed that the time spent by individuals in other residences depends on the proximity to home. However, this assumption may not be applicable to all settings. Therefore, fruitful areas for future research include investigating the effectiveness of perifocal vector control for curtailing chikungunya transmission in settings with socially structured human movement different than the one considered here. In addition, future studies may evaluate the impact of vertical virus transmission within the vector population on the spread of chikungunya and the effectiveness of control strategies for curtailing outbreaks, as well as consider a wide range of *R*_0_ values as some future chikungunya strains may have a higher *R*_0_ value than those considered here.

The early identification of a chikungunya epidemic is a fundamental first step towards implementing effective interventions to rapidly control the disease as well as minimize mortality and morbidity in human populations. Early epidemic identification depends on the efficacy of early warning systems that requires continual and timely surveillance of potential human and mosquito cases, as well as climate drivers for chikungunya transmission. While the control of chikungunya is challenged by case detection, efficacy of vector control and the risk of disease reintroduction from other locations, our model shows that early case detection, combined with perifocal vector control and transmission reduction measures, may be an effective approach for mitigating outbreaks. These findings suggest that such a proactive, well-targeted approach to the containment of chikungunya would complement the traditional vector control measures commonly employed against dengue and other mosquito-borne diseases^55^,^56^,^57^. If chikungunya remains inadequately addressed, the threat that it poses to many regions of the world will likely increase as the mosquito vectors continue to expand geographically.

## Additional Information

**How to cite this article**: Ndeffo-Mbah, M.L. *et al.* Evaluating the effectiveness of localized control strategies to curtail chikungunya. *Sci. Rep.* **6**, 23997; doi: 10.1038/srep23997 (2016).

## Supplementary Material

Supplementary Information

## Figures and Tables

**Table 1:** Parameter definitions and input values.

Parameter	Definition	Values	Refs
*Aedes aegypti*			
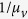	Life expectancy	4–30 days	59
*t*_*incub*_	Incubation period	7–15 days	60,61
*c*	Biting rate of humans by a female mosquito	0.33–1 days^−1^	35,36,38
*β*_*h*_	Transmission rate from human to mosquito per bite	0.30–0.51	62
*β*_*v*_	Transmission rate from mosquito to human per bite	0.06–0.24	estimated*
*Aedes albopictus*			
1/*μ*_*v*_	Life expectancy	8–32 days	59
*t*_*incub*_	Incubation period	2–7 days	61,62
*c*	Biting rate of humans by a female mosquito	0.19–0.39 days^−1^	37,50
*β*_*h*_	Transmission rate from human to mosquito per bite	0.8–1	62
*β*_*v*_	Transmission rate from mosquito to human per bite	0.1–0.36	estimated*
*Human*			
1/*γ*	Incubation period in humans	2–6 days	3,24,63
*χ*	Reduction of mobility for symptomatic cases	50–80%	23,39,40
1/*v*	Pre-symptomatic period	1–3 days	31,32
*κ*	Proportion symptomatic	0.7–0.9	18,63, 64, 65
1/*η*	Infectious period	7–10 days	66, 67, 68
*R*_0_	Basic reproduction number	1.5–7	6,31,43,69

^*^Parameter was estimated by fitting the *R*_0_ of the model against empirical estimates. In the fitting process, *β*_*v*_ was varied from 0.001–1.
