# Supplementary material for: Evaluating the effectiveness of localized control strategies to curtail chikungunya
Source: Sci Rep. 2016 Apr 5;6:23997. doi: 10.1038/srep23997 (PMC4820747; doi:10.1038/srep23997)

Supplementary Figures:

Evaluating the effectiveness of localized control strategies to curtail chikungunya

Martial L. Ndeffo Mbah[1¶*], David P. Durham[1¶], Laura A. Skrip[1], Elaine O. Nsoesie[2], John S. Brownstein[2], Durland Fish[1], and Alison P. Galvani[1, 3]

1 Department of Epidemiology of Microbial Disease, Yale School of Public Health, New Haven, CT, USA

2 Children's Hospital Informatics Program, Boston Children's Hospital, Boston, Massachusetts, USA

3 Department of Ecology & Evolutionary Biology, Yale University, New Haven, CT, USA

Figure S1. R_0 calculation. The model proceeds by A) seeding an initial human exposed to Chikungunay (bold) who then develops infection, potentially B) infecting one or more mosquitoes. C) These mosquitoes then may infect additional humans, who D) may infect additional mosquitoes. As the model is seeded by a single human infection, R_0 is calculated as the number of secondary human infections (C) resulting from primary mosquitoes infections (B).

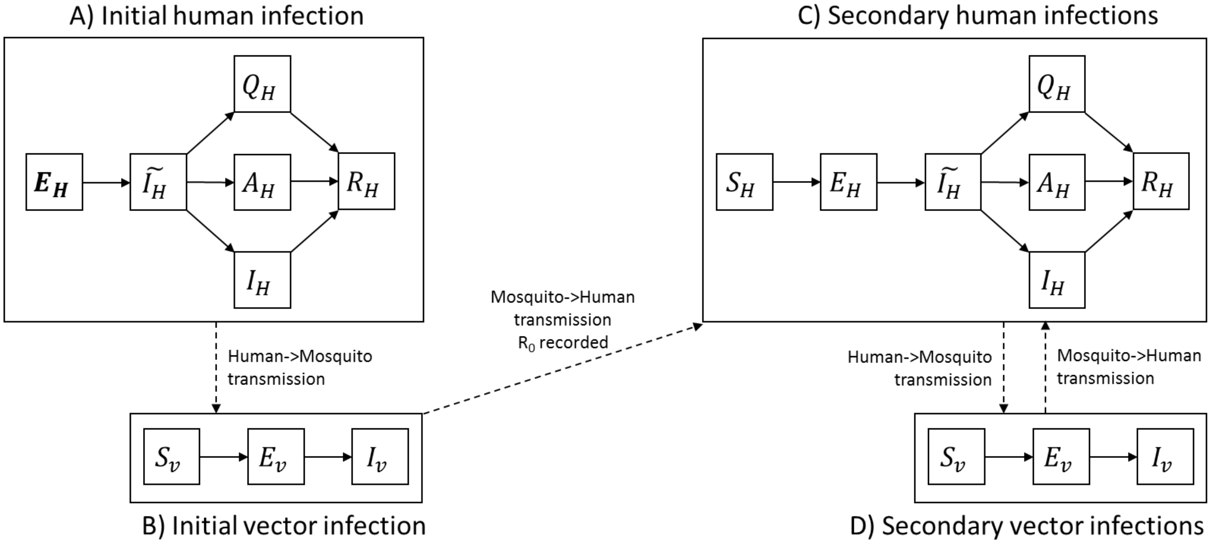

Figure S2. Model flowchart. Model events were stochastically chosen following the Gillespie Algorithm. Events included epidemiological transitions for humans and for mosquitoes, mosquito movement between houses, mosquito birth, and mosquito death.

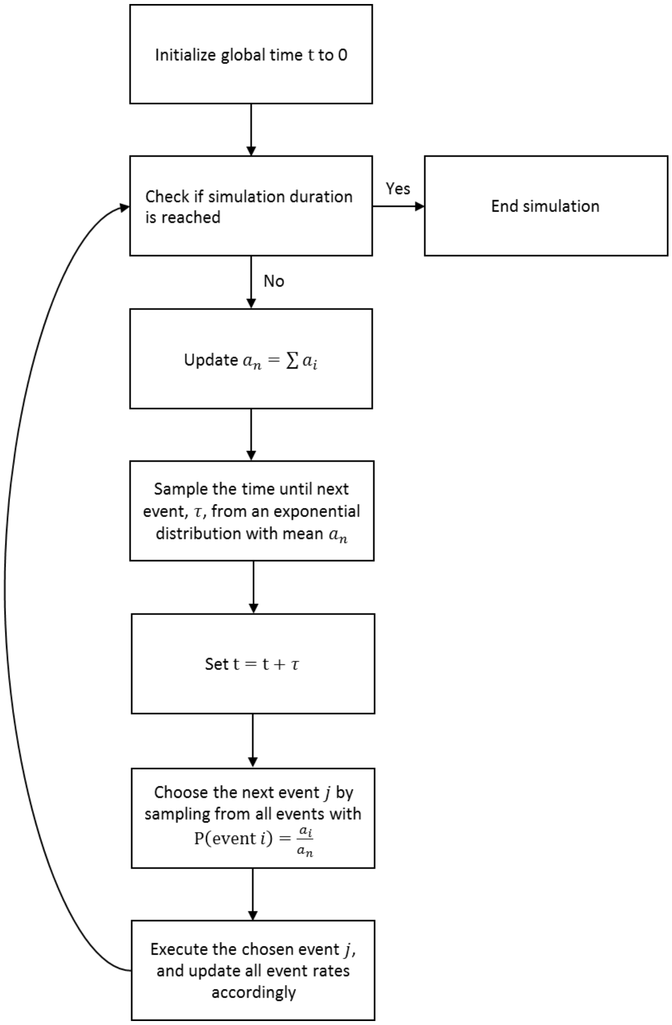

Supplement: Supplementary Information [file srep23997-s1.pdf]
